# OFP1 Interaction with ATH1 Regulates Stem Growth, Flowering Time and Flower Basal Boundary Formation in *Arabidopsis*

**DOI:** 10.3390/genes9080399

**Published:** 2018-08-06

**Authors:** Liguo Zhang, Lili Sun, Xiaofei Zhang, Shuquan Zhang, Dongwei Xie, Chunbo Liang, Wengong Huang, Lijuan Fan, Yuyan Fang, Ying Chang

**Affiliations:** 1 Heilongjiang Academy of Agricultural Sciences, Harbin 150086, China; zlg86@aliyun.com (L.Z.); yinyanlily@163.com (L.S.); zhaoyling1977@163.com (S.Z.); xiedongwei23@126.com (D.X.); liangchunbo2013@163.com (C.L.); huangwengong1736@163.com (W.H.); zxf6216@163.com (L.F.); fayuyanyan@163.com (Y.F.); 2 College of Life Science, Northeast Agricultural University, Harbin 150030, China; 3 College of Mathematics and Information Sciences, Guangxi University, Nanning 530004, China; zhangxiaofei@gxu.edu.cn

**Keywords:** OFP1, ATH1, interaction, stem elongation, flowering, boundary

## Abstract

Ovate Family Protein1 (OFP1) is a regulator, and it is suspected to be involved in plant growth and development. Meanwhile, Arabidopsis Thaliana Homeobox (ATH1), a BEL1-like homeodomain (HD) transcription factor, is known to be involved in regulating stem growth, flowering time and flower basal boundary development in *Arabidopsis*. Previous large-scale yeast two-hybrid studies suggest that ATH1 possibly interact with OFP1, but this interaction is yet unverified. In our study, the interaction of OFP1 with ATH1 was verified using a directional yeast two-hybrid system and bimolecular fluorescence complementation (BiFC). Our results also demonstrated that the OFP1-ATH1 interaction is mainly controlled by the HD domain of ATH1. Meanwhile, we found that ATH1 plays the role of transcriptional repressor to regulate plant development and that OFP1 can enhance ATH1 repression function. Regardless of the mechanism, a putative functional role of ATH1-OFP1 may be to regulate the expression of the both the *GA20ox1* gene, which is involved in gibberellin (GA) biosynthesis and control of stem elongation, and the *Flowering Locus C* (*FLC*) gene, which inhibits transition to flowering. Ultimately, the regulatory functional mechanism of OFP1-ATH1 may be complicated and diverse according to our results, and this work lays groundwork for further understanding of a unique and important protein–protein interaction that influences flowering time, stem development, and flower basal boundary development in plants.

## 1. Introduction

*Arabidopsis* Ovate Family Proteins (AtOFPs), a family of novel transcriptional regulatory proteins, controls multiple aspects of plant growth and development in various plants [1,2,3,4]. Ovate Family Protein1 (OFP1) plays an important regulatory role and was the first characterized member of the Ovate Family Proteins (OFPs). In past reports, OFP1 was shown to be a transcriptional repressor that can regulate cell elongation by controlling *AtGA20ox1*, an important gene involved in gibberellin (GA) biosynthesis [5], in addition to its other functions, such as regulating hypocotyl length and leaf shape, etc. [1,5]. However, because OFP1 lacks predicted DNA-binding domains [5], OFP1 functions must rely on interactions with other proteins for regulation of relevant gene targets. TALE proteins (3-amino acid loop extension homeodomain proteins), including BELL and KNOX transcription factors, potentially interact with OFP1 protein [6] to form complexes that can control multiple plant morphologic characteristics, such as leaf shape, stem elongation, transition from vegetative to reproductive state, etc. [7,8,9,10]. However, few studies described OFP interactions with TALE proteins except a preliminary interaction network of OFP–TALE *proteins* using a large-scale yeast two-hybrid system, however, only the protein interactions of OFP4–KNAT7, BLH3–OFP1, and BLH1–KNAT3–OFP5 were verified in matrix [6,11,12,13,14], and many protein interactions have been subsequently shown to be invalid in interaction network [6,13].

Arabidopsis Thaliana Homeobox (ATH1) belongs to the group of BELL proteins and is an important regulatory protein during plant morphological development [15,16,17,18,19,20]. Notably, of the 18 or so known OFP member genes, ATH1 has only been demonstrated to interact with OFP1, while OFP1 has been shown to control many diverse developmental and growth processes and targets multiple effectors in plants [6]. ATH1 is known to be involved in regulating stem growth, flowering time and flower basal boundary development in *Arabidopsis* [6,17,19,20], ATH1 can regulate shoot apical meristem (SAM) by interacting with the KNOX TALE homeodomain (HD) protein STM [15], and the conjunction of ATH1 and BLADE–ON–PETIOLE can regulate *Arabidopsis* inflorescence architecture, etc. [16]. Previous large-scale yeast two-hybrid studies suggest that ATH1 possibly interact with OFP1 [6], and due to its lack of a DNA-binding domain [5], OFP1 likely plays an important developmental regulatory role via protein–protein interactions with ATH1 in plants, however, the interacted regulation of OFP1 with ATH1 is yet unverified.

Here OFP1-ATH1 protein complex formation was verified using a directional yeast two-hybrid system and bimolecular fluorescence complementation (BiFC). Next, it was shown that the HD protein domain of ATH1 is essential for its interaction with OFP1. Furthermore, ATH1 transcription repression function was revealed using a protoplast transfection system. Because OFP1-ATH1 can regulate flowering time, stem elongation, and flower basal boundary development, analysis was conducted of numerous mutant or transgenic lines, including *Atath1*, *Atofp1*, *Atath1 Atofp1*, *At35S:ATH1*, *At35S:OFP1*, *At35S:ATH1/ofp1*, and *At35S:OFP1/ath1* lines. Moreover, both the *GA20ox1* gene that encodes a GA biosynthetic enzyme [5,13], and the *Flowering Locus C* (*FLC)* gene that influences flowering [19], are possibly regulated by the ATH1-OFP1 complex. Therefore, the interaction of ATH1 with OFP1 is an important protein–protein interaction for regulating growth and development of plants and sheds new light on the OFP-TALE interaction model and its biological function.

## 2. Materials and Methods

### 2.1. Plant Materials

All wild type, mutant, and transgenic *Arabidopsis* plant lines were in the genetic background of ecotype Columbia (Col-0). Seeds were germinated in 1/2× Murashige and Skoog medium (Sigma-Aldrich, St. Louis, MO, USA) and grown in 14 h light, 10 h dark photoperiod cycle at 22 °C, except where otherwise noted.

SALK-113353, a transfer DNA (t-DNA) insertion mutant of *ath1* which is located in exon 4, immediately after the start of the homeobox, was obtained from the ABRC (*Arabidopsis* Biological Resource Center, Ohio, OH, USA). A homozygous mutant of *Atath1* was created and confirmed using special PCR primers of *ATH1* and a t-DNA (File S1). The t-DNA insert site location was confirmed using DNA sequencing. The double mutant of *Atath1 Atofp1* was gained by crossing of *Atath1* and *Atofp1.*

Transgenic plants were obtained via DNA transformation using a floral-dip method using plants with a wild-type genetic background [21,22]. The *35S:AtOFP1* was obtained in our previous work [5], the *35S:AtATH1* used also the same construct method [5]. The three or more transgenic lines with similar phenotypes for all transgenic plants were generated, T3 generations were identified as stable using phenotypic analysis of progeny. Meanwhile, *At35S:ATH1*/*ofp1*, *At35S:OFP1*/*ath1* and *Atath1 ofp1* plants were created by crossing existing mutants and served as additional tools for elucidation of the roles of OFP1 and ATH1 in plant development. Five gene accession codes, AT4G32980 (*ATH1*), AT5g10140 (*FLC*), At5G01840 (*OFP1*), AT4G25420 (*GA20ox1*), AT4G22570 (*APT*) were used in this work.

### 2.2. Plasmid Construction and Yeast Two-Hybrid System Testing

The open reading frames of *OFP1* and *ATH1* were cloned from wild-type *Arabidopsis*. The HD protein domain and SKY-BELL protein domain of ATH1 were compartmentalized based on the method of [19] using the online tool http://smart.embl-heidelberg.de/. DNA fragments containing the HD and SKY-BELL domain of ATH1 were obtained via PCR using primers to those respective domains using *ATH1* cloned in pUC18 plasmid vector as template (File S1). Amplified *ATH1* HD and SKY-BELL fragments were cloned into expression plasmids that were transformed into yeast Y2HGold lines and ATH1 complementary DNA (cDNA) was cloned into vector pGADT7. Yeast cells were cultured in synthetic defined quadruple dropout (SD QDO) medium lacking three amino acids and adenine (-Leu, -His, -Trp, -Ade) after the yeast cells had been earlier selectively cultured in SD Double Dropout (SD DDO) medium lacking only two amino acids (-Trp, -Leu). The negative control used pGBKT7-Lam co-expressed with pGADT7-SV40 and the positive control employed proteins co-expressed in the same yeast cells from separate plasmids, pGBKT7-p53 and pGADT7-SV4. Control protein–protein interactions in the positive control had been verified in a previous report [12], three replicates were used in the assays.

### 2.3. Bimolecular Fluorescence Complementation

Yellow fluorescent protein C-terminal fragments and N-terminal fragments were separately fused to OFP1 and ATH1, respectively, to form fragments coding for expression of OFP1-YFPC and ATH1–YFPN [23], respectively. Cloned ATH1 and OFP1 cDNAs were separately sub-cloned into the pSAT6–cEYFP–N1 vector and pSAT6-nEYFP-N1 vector, respectively, and *Arabidopsis thaliana* protoplasts were transfected with these vectors using polyethylene glycol transfection [24]. A negative control (OFP1–YFPC and RACK1–YFPN, RACK1-receptor for activated C kinase 1) was used [5]. Fluorescent images were analyzed using a Leica TCS SP2 Laser Scanning Confocal Microscope (Leica Microsystems, Buffalo Grove, IL, USA).

### 2.4. Transient Transfection of Protoplasts and Plasmid Construction

Two types of effector plasmids and a reporter plasmid were constructed then co-transfected into protoplasts. To generate effector plasmid constructs, *35S:GD-ATH1(OFP1)* construct cloned into pUC19 was used as template to amplify *OFP1*-derived PCR products that were then cloned into pUC19 to create constructs with two 35S enhancer CaMV promoters upstream of PCR products [25]. PCR products were inserted so that their protein coding sequences were in-frame with GD (Gal4 DNA-binding domain) or HA (hemagglutinin) tags [25,26]. The final pUC19 plasmid-based effector constructs were digested with EcoRI and the inserts ligated into the binary vector pZP211 [27]. A second effector plasmid contained a herpes simplex virus transactivator domain (LD-VP16). The reporter gene binary vector construct contained LexA(2X)-Gal4(2X):GUS, where GUS designates *β*-glucuronidase. Both effector constructs and reporter vector were purified using an EndoFree Plasmid Maxi Kit (Takara, Dalian, China) and were co-transfected into protoplasts using previously reported methods [26,28]. Experimental data were statistically analyzed by *t*-test. The transfection operation incorporated at least two technical replicates and three biological replicates.

### 2.5. Expression Assay of ATH1, Scanning Electron Microscopy and Cell Length

Total RNA was extracted from *Arabidopsis* tissues using the instructions with the RNA extraction kit (Takara). The real time PCR (qPCR) assay was carried out according to the manufacturer’s instructions using a qPCR kit (Takara). Relative transcript levels of *GA20x* and *FLC* were compared between wild-type and either transgenic lines or mutant lines. Expression pattern of *ATH1* and *OFP1* were also examined in different wild type tissues (from four days to flowering phase). The adenine phosphoribosyltransferase gene (*APT*) of *Arabidopsis thaliana* was used as a constitutive control [20]. The assay was performed in triplicate. The fluorescent dye, iQ^TM^ SYBR^®^ Green Supermix (Bio-Rad, Hercules, CA, USA), was used to perform the real-time PCR assay. Fluorescence was measured using an iQ5 Multicolor Real-Time PCR Detection System (Bio-Rad). The relative expression value was calculated by the 2^−ΔΔCt^ method. The reaction was performed in parallel using three biological replicates and three technical replicates. The numerical analysis generated Ct values. The *Actin1* gene (*ACT1*, At2g37620) served as a positive control in reverse transcription PCR.

ATH1 promoter which is 812-bp DNA fragment of *ATH1* coding region upstream, which was fused to the GUS reporter gene for generating prom *ATH1*:GUS transformation plants, and the construction method is same as previous report [16]. Histochemical staining was used to examine the GUS activity using substrate 5-bromo-4-chloro-3-indolyl β-d-glucuronide. The general procedure of histochemical staining for GUS activity has been described in past reports [5,12]. The GUS activities of seedlings at various developmental stages and of organs from adult plants were examined.

The assay procedure using scanning electron microscope was developed by Gómez-Mena method [20] and 10–15 plants of at least three lines were analyzed. Plants were fixed in 2.5% glutaraldehyde in phosphate buffered saline at 4 °C overnight, dehydrated in an ethanol series, and critical-point dried in liquid CO_2_. For cryo-scanning electron microscopy, flowers were frozen in nitrogen slush at 190 °C. Ice was sublimated at −90 °C, the Philips XL 30 FEG was used in assays.

The cell lengths were measured in 10-day-old, light-grown seedlings using a dissecting microscope. The hypocotyl epidermal cells were visualized using a Leica DM-6000B upright microscope (Leica, Solms, Germany) with phase and digital image acquisition equipped with a Leica FW4000 (Leica) digital image acquisition and processing system.

## 3. Results

### 3.1. Detection of Protein Interaction between ATH1 and OFP1

Data of previous reports [6] suggest that BEL1-like HD transcription factors may interact with OFPs as part of a putative complicated protein–protein interaction network. In this model, a BEL1-like protein is putatively regulated through interactions with several OFPs members. For example, one BEL1-like protein, BLH6, was purported to interact with OFP1, OFP4, and OFP5. Meanwhile, another BEL1-like protein, BLH10, was thought to interact with OFP1, OFP2, OFP4 and OFP5. However, the only putative ATH1 interaction has been suggested to occur with OFP1 and may be part of a tentative TALE-OFP interaction network.

Here, the interaction of ATH1 with OFP1 was detected using a targeted yeast two-hybrid test, rather than a large-scale preliminary screening.

**Figure 1:**
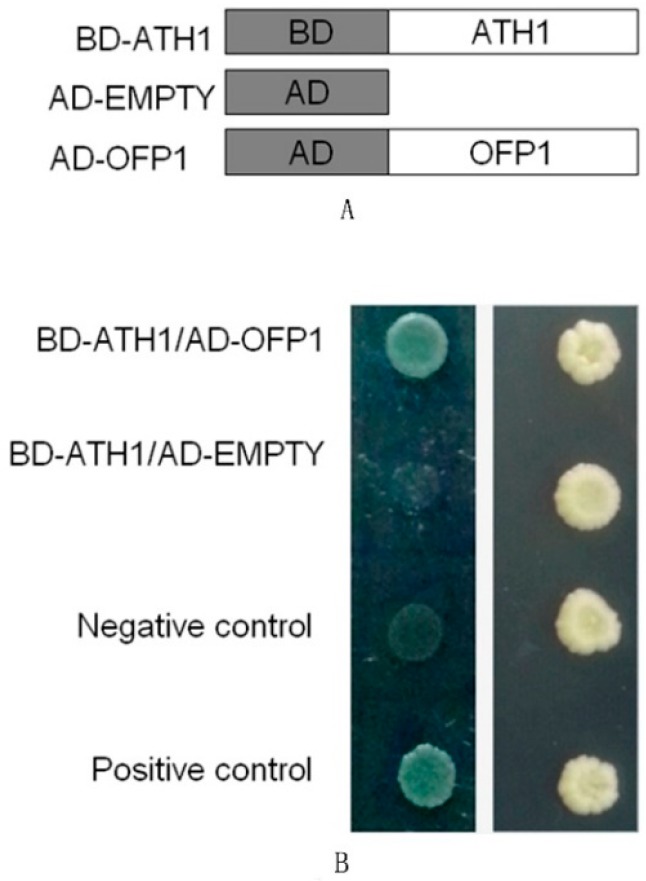
Yeast two-hybrid results showing OFP1–ATH1 interactions. (**A**) Diagram of constructs used in yeast two-hybrid assays. (**B**) ATH1 interacts with OFP1 in yeast cells. The protein interaction was detected on synthetic defined quadruple dropout (SD QDO) medium (left) by measuring yeast cell growth state after the yeast cells had been screened and had positive growth on SD double dropout (SD DDO) medium (right) (QDO lacks His, Leu, Ade, and Trp; DDO lacks Trp and Leu). BD, DNA-binding domain; AD, activation domain.

The results of yeast two-hybrid studies show that ATH1 can adequately interact with OFP1 after fusion proteins OFP1-AD (OFP1–activation domain) and ATH1-BD (ATH1–DNA binding domain) were both co-expressed in yeast cells (Figure 1). A negative control (pGBKT7-Lam and pGADT7-SV40) and a positive control (pGBKT7-p53 and pGADT7-SV4) were included in the experiment (Figure 1, top). These results are consistent with results of a previous report [6] that suggested a possible interaction between ATH1 and OFP1 in vivo. However, it is not clear which protein domain of ATH1 interacts with OFP1 protein, though the OVATE domain of OFP might interact with a TALE protein, as demonstrated in previous reports [6].

**Figure 2:**
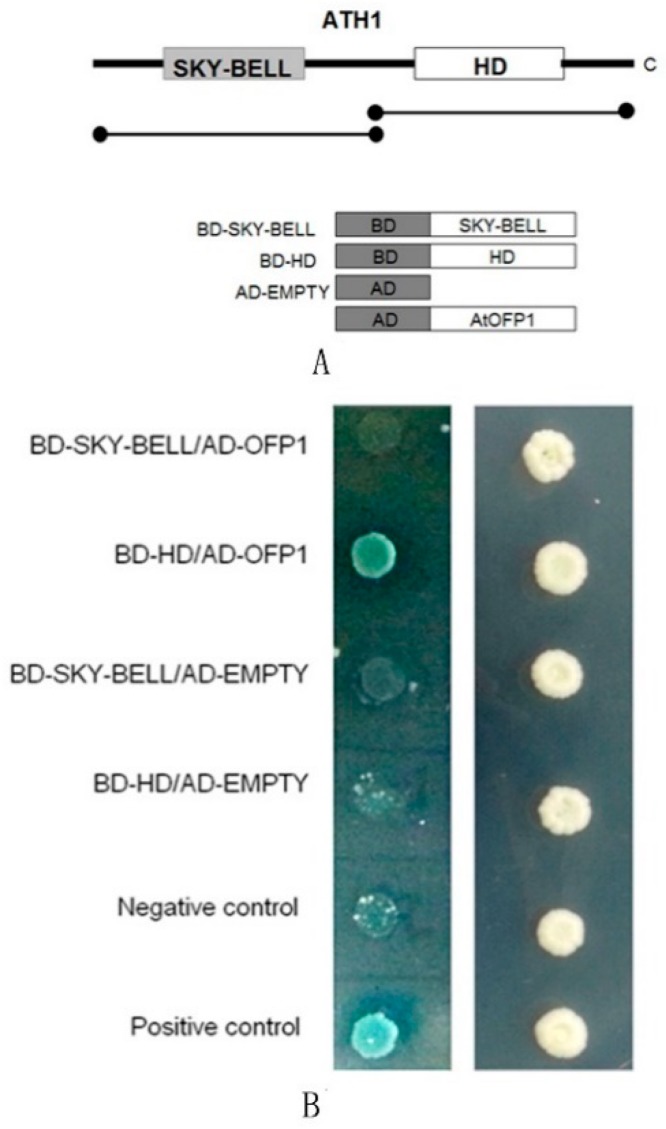
Verifying the specific protein domain of ATH1 that interacts with OFP1 Protein. (**A**) Representation of ATH1 structure showing SKY-BELL domain and homeodomain (HD). (**B**) Yeast hybrid result showing that fragments of BD–ATH1 interact with AD–OFP1 in yeast cells.

The two protein-interaction domains of ATH1 studied here, HD and SKY-BELL, were each separately fused to BD and expressed in yeast to ascertain whether either ATH1 domain can interact with OFP1. The result demonstrates that the ATH1 HD domain can interact with integrated OFP1 protein, while the SKY-BELL domain of ATH1 does not interact with OFP1 (Figure 2).

**Figure 3:**
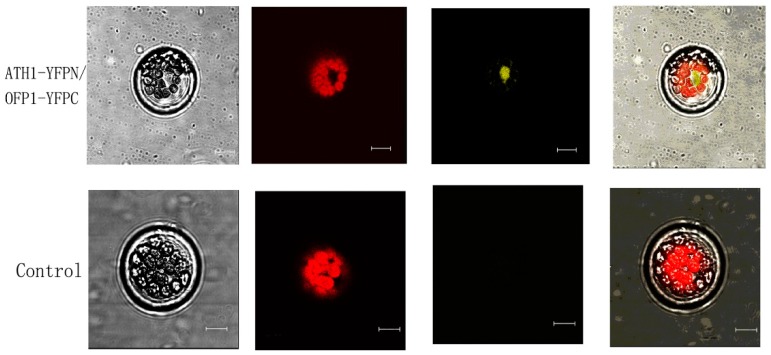
Bimolecular fluorescence complementation (BiFC) Monitoring of the protein interaction between ATH1 and OFP1. The expression of OFP1–YFPC and ATH1–YFPN after transfection into *Arabidopsis* protoplasts. The yellow fluorescence shows that functional YFP was formed as a result of the interaction of ATH1 with OFP1. Bar = 10 μm. Bottom: Negative control (OFP1–YFPC and RACK1–YFPN) is shown. Bar = 10 μm.

The BiFC method was used to further verify the presence of an ATH1 interaction with OFP1 in vivo. Co-expression of OFP1 fused to C-terminal YFP (OFP1–YFPC) and ATH1 fused to N-terminal YFP (ATH1–YFPN) was achieved in an *Arabidopsis* protoplast transient expression system [28]. Yellow fluorescence was observed after YFPC was brought into close proximity with YFPN through OFP1–YFPC binding to ATH1–YFPN via a binding interaction between OFP1 and ATH1. The negative control (OFP1–YFPC and RACK1–YFPN) employed a parallel co-expression system of non-interacting proteins in place of ATH1 and OFP1 [29]. The data shown in Figure 3 show that OFP1 can interact well with ATH1 in vivo. The other negative control (Figure S1, Bar = 10 μm.), ATH1 and OFP4 (Figure S1A), ATH1–YFPN—with empty pSAT6-cEYFP-N1 (Figure S1B), OFP1–YFPC with empty pSAT6-nEYFP-N1 (Figure S1C), and positive control KNAT7 and OFP4 [11] were shown in Figure S1D.

### 3.2. ATH1 Functions as a Transcriptional Repressor

**Figure 4:**
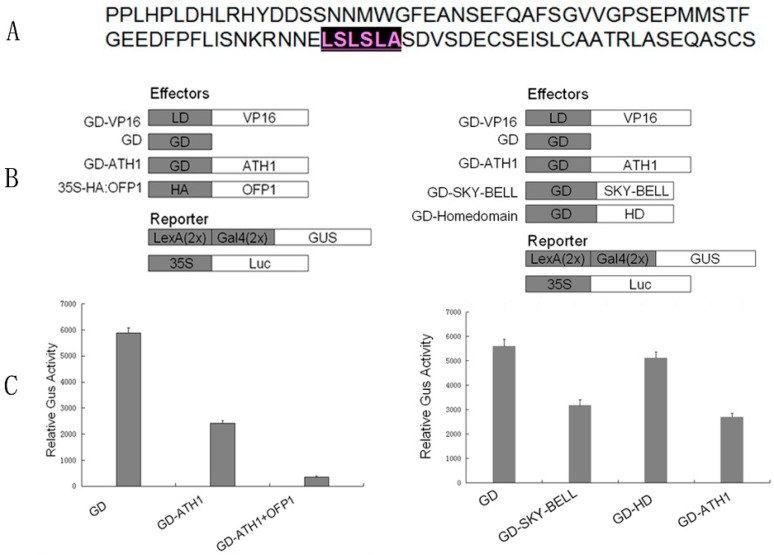
Sequence analysis results and transcription activation evaluation. (**A**) A classical motif (highlighted) was found in the amino acid sequence of ATH1. Both effector and reporter constructs were used in the transfection assays. (**B**) Transcriptional repression of ATH1 was identified and its repression function was increased by ATH1 interaction with OFP1 in vivo. The expression of 35S:luciferase (Luc) was used to normalized the expression of the GUS reporter gene. (**C**) The protein domain involved in ATH1 repression activation was identified by using a protoplast transfection system for checking relative GUS activity. LD, LexA DNA-binding domain; GD, Gal4 DNA-binding domain; HA, hemagglutinin.

Previous reports have demonstrated that OFP1 is a transcriptional repressor [5,12]. However, it is not clear whether ATH1 can also repress transcriptional activity. A classical transcriptional domain (LSLSLA, where L is leucine, S is serine, and A is alanine) has been observed after analysis of the ATH1 amino acid sequence (Figure 4). A protoplast transfection system was next employed to determine if ATH1 exhibits transcriptional repression [5,30]. The results are displayed in Figure 4.

The GD-ATH1 (or OFP1) and LD-VP16 were created, whereby GD corresponds to the Gal4 DNA-binding domain and LD corresponds to the LexA DNA-binding domain, respectively. LexA and Gal4 have been demonstrated to function as potent effectors in a LexA-Gal4-GUS reporter system. The experimental stability of this system has been verified in past reports and this system has been successfully used for evaluation of transcriptional repressors KNAT7 [30], BLH6 [11], BLH3 [12], and AUX/IAA proteins [31,32].

In our investigation, the GUS gene served as a reporter gene which was controlled by DNA-binding domain sites of LexA and Gal4. Next, the reporter plasmid LexA(2X)-Gal4(2X):GUS and the two types of effector plasmids were co-transfected into protoplasts. The effector plasmid LD–VP16 contained a chimeric protein consisting of LexA and DBD fused to the VP16 transcriptional activation domain of herpes simplex virus for which transcription was driven by the CaMV 35S promoter. A second effector plasmid contained a protein consisting of the Gal4 DBD fused to the gene of interest, for example GD–ATH1, for which transcription was also controlled by the CaMV 35S promoter. A 35S-driven expression of GD domain alone was used as a control for comparison to the second effector plasmid.

The results show an obvious downregulation of GUS expression level by co-transfection of GD–ATH1, GD–OFP1, and LD–VP16 effectors. (Figure 4). The results also reflect that ATH1 is a transcriptional repressor, not an activator.

Meanwhile, fusion proteins GD–SKY–BELL and GD–HD were expressed to detect activity of an ATH1 repression domain (Figure 4). The result implies that GD-SKY–BELL is involved in transcription repression function, with a level of repression comparable to that of GD-ATH1. In addition, the results also show that the HD domain of ATH1 is not involved in transcription repression activity.

### 3.3. Expression Pattern of ATH1 and OFP1

**Figure 5:**
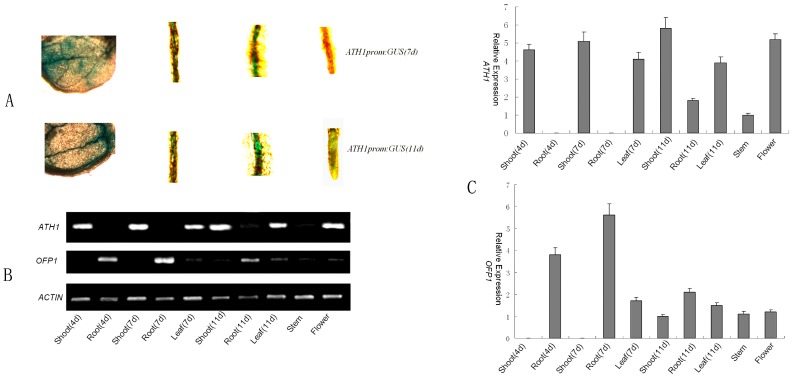
*ATH1* and *OFP1* expression in *Arabidopsis*. (**A**) *ATH1prom*: GUS expression in *Arabidopsis* seedlings at 7 days and day 11 from left to right: leaf, hypocotyl, closeup view of root, and closeup view of root tip. (**B**) The expression pattern of *ATH1* and *OFP1* were examined in different wild-type tissues by using real tiem PCR (qPCR). (**C**) *ATH1* expression in various organs in various developmental phases detected by reverse trancription PCR (RT-PCR). *ACT1* (actin gene) was used as a control for loading.

The expression pattern of *ATH1* and *OFP1* were examined in different wild-type tissues by using qPCR and reverse trancription PCR (RT-PCR). *ATH1 and OFP1* expression features are shown in Figure 5. Similar *ATH1*/*OFP1* expression profiles were observed at various time points in shoots, roots, leaves, hypocotyls, whole stem and flower buds, but not of shoot and root in 4-day-old and 7-day-old seedlings. (Figure 5), suggest that expression of *ATH1* and *OFP1* was involved in developmental regulation.

### 3.4. Role of OFP1 and ATH1 in Plant Development

**Figure 6:**
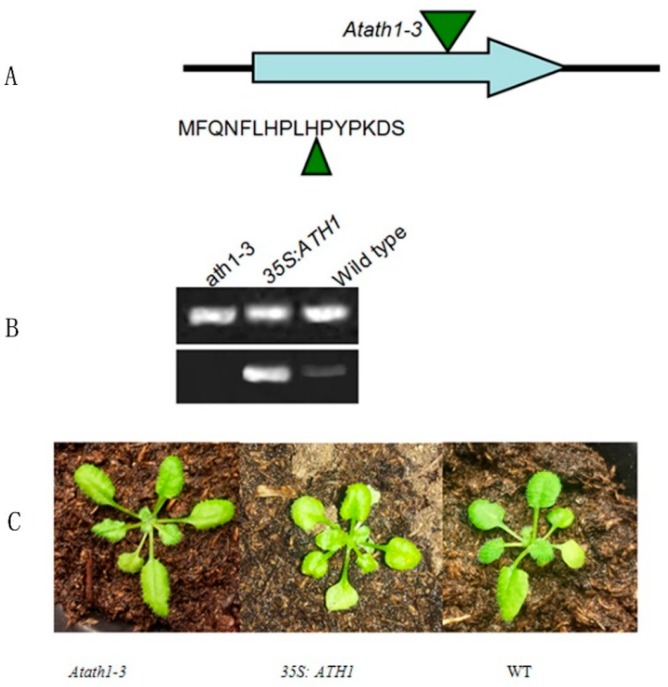
*Atath1-3* transfer DNA (t-DNA) insertion mutant characteristics. (**A**) Location of t-DNA insertion in *ATH1* sequence of exon [33]. (**B**) *Atath1-3* lacks *ATH1* transcription as shown using RT-PCR (top row shows positive control *ACT1*, bottom row *ATH1*). (**C**) Phenotypes of *Atath1-3*, *At35S:ATH1*, and wild type (WT) are shown.

The phenotypes of *ath1* and *ofp1* mutant plants were analyzed to explore OFP1 function as part of the ATH1-OFP1 protein complex. The *Atofp1-1* mutant had been obtained in a previous study [5]. SALK_113353, an *ath1-3* mutant t-DNA insertion line was obtained from the ABRC [20,33]. In this work, an insertion exon region mutant obtained by screening and confirmed by DNA sequencing lacked a complete *ATH1* gene and had been selected for screening after RT-PCR did not amplify *ATH1*; this mutant was designated *Atath1-3* (Figure 6).

**Figure 7:**
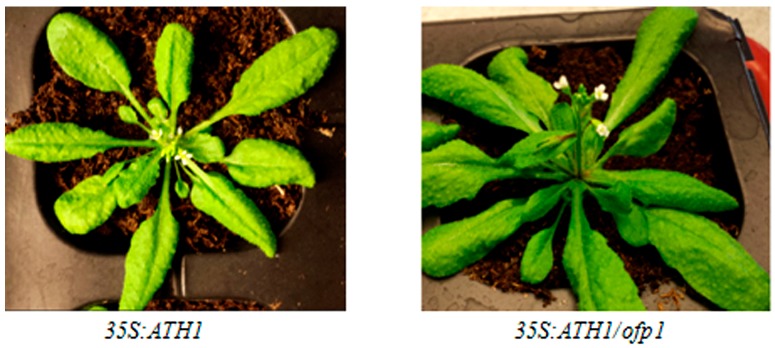
Phenotypes of *At35S:ATH1* and *At35S:ATH1/ofp1* lines during the flowering Stage. *At35s:ATH1, At35s:ATH1*/*ofp1*, *Atath1 Atofp1* and other lines are shown in Figure S2.

*At35s:OFP1* lines manifested pleiotropic phenotypes (Figure S2). Phenotypes of *At35s:ATH1* and *At 35s:ATH1*/*ofp1* are shown (Figure 7). *At35s:ATH1* exhibited extremely short stems at the flowering stage, while the stem length of *At35s:ATH1*/*ofp1* was significantly longer compared to *At35s:ATH1.* Meanwhile, plant phenotypes of *At35s:OFP1*/*ath1*, *Atath1*, *Col*, *Atofp1* and *Atath1 Atofp1* are listed in Figure S2.

Detailed morphologies averaged for 6 to 10 plants of each of the seven lines were extensively surveyed using the method of Cole [19] (Table 1).

The *Atath1*, *Atofp1* and *Atath1 Atofp1* mutants both exhibited early flowering and decreased leaf number as compared to wild-type plants, with earlier flowering by five days, two days and six days, respectively, and decreased leaf quantity by about four leaves, two leaves and four leaves, respectively. At the same time, *At35S:ATH1* and *At35S:OFP1* plants exhibited contrasting phenotypes compared to those of *Atath1* and *Atofp1* plants, exhibiting later flowering by six days and about two days, respectively, and increased leaf quantity by about three leaves and one leaf, respectively. Meanwhile, *At35S:OFP1* plants manifested other obvious pleiotropic phenotypic traits compared to *At35S:ATH1* plants, such as circular and curving leaves and reniform cotyledons [5] (Figure S2). At the same time, *At35S:ATH1*/*ofp1* plants and *At35S:OFP1*/*ath1* plants exhibited later flowering by four days and two days, respectively, and increased leaf quantity by about three leaves and about one leaf, respectively, compared to wild plants. Furthermore, *At35S:ATH1*/*ofp1* and *At35S:OFP1*/*ath1* plants exhibited increases in leaf quantity of two more leaves and one leaf, respectively, and earlier flowering by four days and two days, respectively, compared to wild type. Collectively, differences in flowering time and leaf quantity of mutants exhibited significant deviations from wild-type, as demonstrated using F-test (Table 1). Furthermore, the difference of inflorescence height is highly significant among different genetics materials. However, only the *At35S:ATH1* show significant difference compared to wild plants, *Atofp1* and *Atath1* do not show significant difference, respectively, compared to *At35S:ATH1*/*ofp1* plants and *At35S:OFP1*/*ath1* plants. The *At35S:ATH1* and *At35S:OFP1* plants also do not show significant difference, respectively, compared to *At35S:ATH1*/*ofp1* plants and *At35S:OFP1*/*ath1* plants (Table 1).

**Figure 8:**
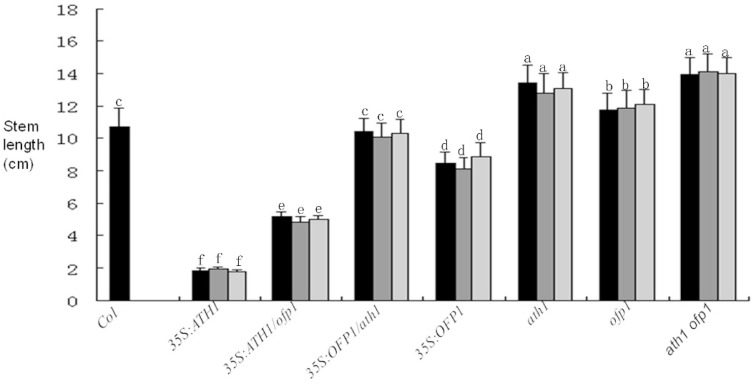
Comparison of stem lengths after flowering for 10 days. Three distinct lines were analyzed for each mutant type, with significant differences from different genetics materials marked with different letters (*p* < 0.05). The error bars reflect standard deviations of various plant lines compared to wild-type. The 10–15 plants were analyzed after flowering for 10 days.

In previous reports, the stem growth of *35S:ATH1* plants was inhibited [19]. However, these results did not indicate whether OFP1 played a role in regulating stem growth as part of the OFP1-ATH1 protein complex. Here the hypocotyl cell lengths of three independent lines for each genetic mutant type. The results show that the hypocotyl cell length of *At35S:ATH1* plants was suppressed, while the hypocotyl cell length of *At35s:ATH1*/*ofp1* was significantly longer compared to *At35s:ATH1*. At the same time, *Atath1*, *Atofp1* and *Atath1 Atofp1* plants showed significantly longer stems compared to wild-type. Furthermore, the hypocotyl cell length of *At35S:OFP1*/*ath1* plants was similar to wild-type, while *At35S:OFP1* plants exhibited slightly shorter hypocotyl cell length (Figure 8).

**Figure 9:**
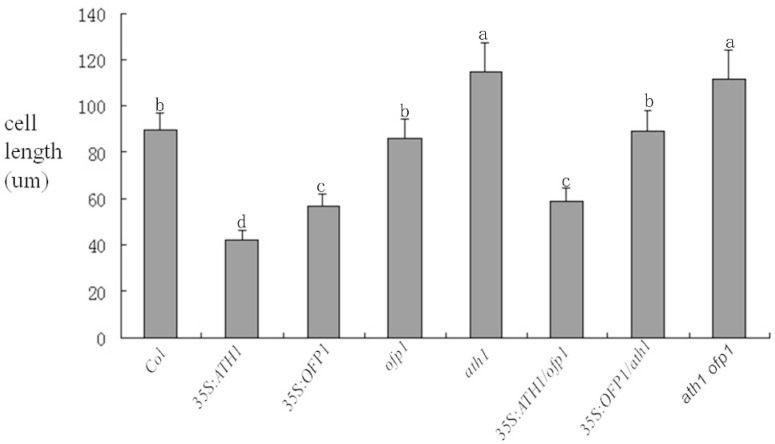
Lengths of hypocotyl epidermis cells in various mutants (10-day-old seedlings). Different letters represent significant differences among different genetics materials (*p* < 0.05). The bars represent standard deviations. The 7–10 plants of each genetic type, and a least 10 cells of each plant were analyzed.

Stem length is mainly controlled by alterations in either cell quantity or length. In past reports [5,19], *At35S:OFP1* and *At35S:ATH1* could repress cell elongation; And OFP1 can interact with ATH1 however, the relative roles played by ATH1 and OFP1 within OFP1-ATH1 complexes in regulating cell elongation are not known. Our results indicate that cell lengths of *Atath1* and *Atath1 ofp1* lines were increased compared to wild-type. Notably, the lengths of *At35S:ATH1*/*ofp1* plant cells, although obviously decreased compared to wild-type, were significantly increased compared to cells of *At35S:ATH1*. At the same time, *Atofp1* and *At35S:OFP1*/*ath1* cell lengths were not significantly different from wild-type (Figure 9).

**Figure 10:**
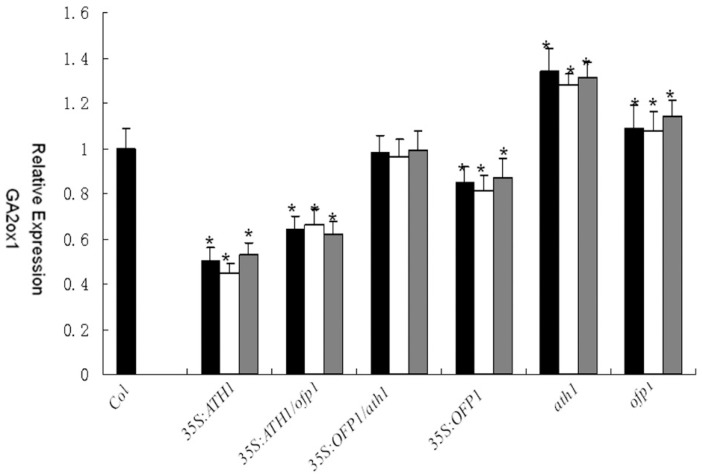

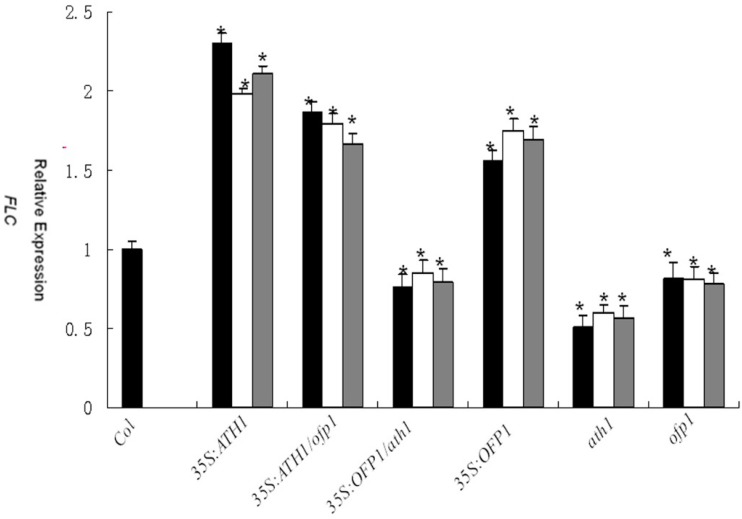
Analysis of relative expression levels of *GA20ox1* and *FLC* by using quantitative PCR (qPCR) of three transgenic lines of each genotype in 14-day-old seedlings. * indicates significantly different (*p* < 0.01). The bars represent standard deviations. Data were analyzed using the 2^−ΔΔCt^ method and normalized using the expression of adenine phosphoribosyltransferase (APT).

Previous research has showed that the expression of *ATH1* could activate *FLC*, a flowering repressor [16,33,34,35]. However, it is not known if OFP1 function in regulation of *FLC* expression involves an interaction between OFP1 and ATH1. The real-time PCR results for different lines showed that *FLC* expression levels in *35S:ATH1* and *35S:OFP1* lines were both upregulated. Conversely, the *FLC* expression levels of *ath1, ofp1* and *ath1 ofp1* were downregulated (Figure 10). At the same time, *Ga20x1* a biosynthetic enzyme gene for GA that is known to regulate cell proliferation and elongation could play a role. Notably, *35S:OFP1* lines have been shown to repress expression of *GA20x1* in past reports [5]. However, the mutations of *Atofpl*, *Atath1*, and *At35S:ATH1* on regulation of *GA20x1* expression level are not known. Our data demonstrate that *GA20x1* was downregulated in *At35S:ATH1* lines but upregulated in *Atofp1* and *Atath1* lines using real time PCR (qPCR) (Figure 10).

**Figure 11:**
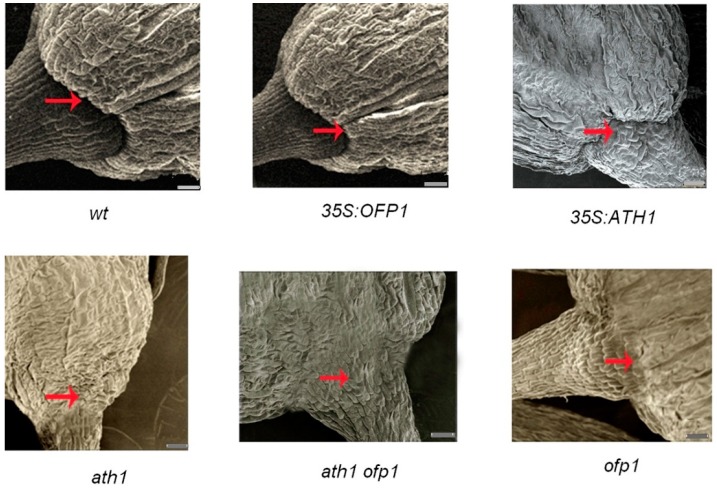
Comparison of basal boundaries using scanning electron microscopy. The mutant *Atath1* and *Atath1 Atofp1* exhibited fused flower basal boundaries and *Atofp1* exhibited partly fused boundaries. Meanwhile, *At35S:OFP1* and *At35S:ATH1,* were similar to WT in their regulation of flower basal boundaries. At least three lines (6–10 plants) were analyzed for each mutant line. The red arrow indicated flower basal boundaries. Bar = 500 µm.

Furthermore, ATH1 appears to be involved in regulating basal boundaries of reproductive and vegetative organs [20]. However, the role of OFP1 in forming organ basal boundaries is not known. Scanning electron microscopy results show that basal boundaries of various organs were not changed in *Atofp1* lines except for possible effects on development of flower basal boundaries (Figure 11). Meanwhile, *Atath1* and *Atath1 ofp1* lines showed fused basal boundaries of flowers while *Atofp1* lines showed partly fused boundaries. Finally, *At35S:OFP1* results were similar to wild-type and *At35S:ATH1* in regulating flower basal boundaries.

## 4. Discussion

Past research has indicated that complex interactions may exist between OFPs with BLHs and (or) KNOXs [6]. Nevertheless, OFP–KNOX–BLH indicates a possible protein complex configuration that may be involved in regulating transcription.

The results described in this study show that ATH1 is a transcriptional repressor with activity similar to that of OFP1. At the same time, the ATH1–OFP1 protein complex is possibly involved in regulation of flowering time and stem growth and control of formation of the flower basal boundary in *Arabidopsis*.

### 4.1. Interaction of OFP1 with ATH1 In Vivo for Repression of Transcription

The OFP1 protein can interact with ATH1 protein in vivo, as shown using yeast two-hybrid and BiFC assays. More specifically, the HD domain of ATH1 possibly plays an indispensable role in the ATH1–OFP1 interaction, as demonstrated using protoplast transfection with various effectors. In past research, the SKY–BELL domain, another important domain of the ATH1 protein, had been shown to be a necessary domain that interacts with KNOX protein to form transcriptional complexes [36,37]. Meanwhile, a complicated network of interactions possibly exists between BELL proteins and KNOX proteins [6]. Therefore, the interaction of OFP1 with ATH1 also possibly includes KNOX protein function, but is likely an indirect protein interaction. Furthermore, the HD domain of ATH1 probably plays an important role in any possible OFP1–ATH1–KNOX protein interaction model.

ATH1 contains a LxLxL sequence which could be a repressor motif. Although the results above show that ATH1 is a transcriptional repressor that can interact with OFP1, transcriptional repression of OFP1 was observed [5]. Although biological functions of ATH1 and OFP1 might overlap in their regulation of flowering time and stem growth due to a common pathway that they share, ATH1-OFP1 protein transcription complexes probably act through protein–protein interactions to regulate plant development.

### 4.2. The OFP1–ATH1 Protein Complex Is Involved in Regulating Flowering Time, Stem Growth, and Flower Basal Boundary Development

Little information is known about complexes between proteins containing the TALE HD and OFP, with the exception of past reports demonstrating OFPs to be components of a putative multiprotein transcription regulatory complex containing BLH6 and KNAT7 [11]. Meanwhile, studies have shown that a KNAT7–OFP4 complex can regulate secondary wall formation in *Arabidopsis* [30], while a protein complex of BLH1–KNAT3–OFP5 is involved in ovule development in *Arabidopsis* [14]. 

In addition to the understood regulatory function of ATH1 in flowering time and stem growth [19], the interaction between ATH1 and OFP1 in vivo indicates that OFP1, as a part of an OFP–BLH protein complex, also regulates these characteristics. Moreover, ATH1 plays a role of transcriptional repressor, as observed for OFP1 using activation assays described above. Meanwhile, ATH1 expression can delay flowering time, as demonstrated for *At35S:ATH1* and *At35S:ATH1*/*ofp1* lines that exhibit later flowering time and produce greater number of leaves compared to wild-type plants. Transcriptional repression of ATH1 is also consistent with a biological function such as repression of flowering. Furthermore, in past reports, OFP1 also interacts with BLH3 in vivo to regulate flowering time, and BLH3 plays the role of transcriptional activator to promote flowering, the activity of BLH3 could be inhibited by OFP1 [12,13]. However, ATH1 functioned as a repressor to delay flowering, and OFP1 can enhance ATH1 repression function. The result implies that OFP1 possibly delay flowering time by interacting with BLH3 to decrease BLH3 transcription activity and interacting with ATH1 to increase ATH1 transcription repression activity.

Conversely, *Atofp1*, *Atath1* and *Atath1 Atofp1* lines showed early flowering but also produced less quantities of leaves, further implying that ATH1 and OFP1 possibly regulate a similar developmental trait, and an additive effect in the double mutant may suggest that they have partial redundant effect and may or may not participate in different pathways that regulate the same developmental trait. However, flowering time and leaf quantity of *At35S:OFP1/ath1* and *At35S:OFP1* were comparable, indicating that OFP1 likely does not depend on ATH1 to exert its regulatory role. However, due to the fact that OFP1 lacks a DNA-binding domain, it must exert transcriptional regulation through interactions with other proteins. Furthermore, the result that the significant difference of inflorescence height and no significant deviation in the plastochron, it implied that the interaction of OFP1 with ATH1 possibly involved in regulating inflorescence height, and did not obviously control plastochron development.

In previous findings, *At35S:ATH1* also exhibited late flowering with all its shades [38], the difference degree of flowering time possibly resulted from different insertion sites of *At35S:ATH1* in genome and different growth conditions, nevertheless, the lines both activate *FLC* in common, but they were not further expounded that how did other proteins regulate ATH1 expression. Notably, *At35S:ATH1* flowered late and produced more leaves compared to *At35S:ATH1*/*ofp1*, indicating that OFP1 might enhance repression of flowering by ATH1. Additionally, protoplast transfection assays demonstrated that OFP1 could increase ATH1 transcription repression function (Figure 4), possibly because OFP1 negatively regulates ATH1 or ATH1-KNOX protein complexes. Meanwhile, as a whole, *OFP1* and *ATH1* exhibited similar expression patterns by using qRT-PCR (Figure 5), implying that OFP1 and ATH1 might be present in the same tissues. However, the expression of OFP1 and ATH1 in root and shoot show fine distinction in four-day-old and seven-day-old seedlings, it implies that both factor possibly have independent roles in the tissues. These results further show that OFP1 can exhibit a regulatory function as a part of a protein complex with ATH1. Nevertheless, OFP1 might depend on other proteins to regulate transcription, due to its lack of a DNA binding domain. In previous reports, *At35S:OFP1* exhibited various morphological changes [5]. However, our results indicated that the phenotype of *At35S:OFP1/ath1* was similar to that of *At35S:OFP1*, thus indicating that OFP1 might not depend upon ATH1 in regulating flowering time. However, the stem length of *At35S:OFP1/ath1* was similar to wild-type, even though the stem growth of *At35S:OFP1* was repressed. These results indicate that OFP1 function might partly depend on ATH1 to regulate stem development in a model similar to that of OFP4, which partly depends on KNAT7 to regulate formation of the secondary cell wall [11,30]. Meanwhile, *Atofp1* exhibits earlier flowering and longer stem lengths. Although OFP family proteins might share overlapping biological functions, the results collectively imply that OFP1 may play an important role in regulating flowering and stem length. Furthermore, the mechanism underlying the regulatory function of OFP1-ATH1 is complicated and diverse due to the lack of a DNA-binding domain in OFP1. And, the trial system of BiFC and two-hybrid system did not eliminate function of other proteins. Therefore, the interaction of OFP1 with ATH1 is probably not direct and may require other proteins. This speculation is further supported by the fact that OFP1 does not depend on ATH1 to regulate flowering time, but OFP1 possibly partly depends on ATH1 to regulate stem elongation. Indeed, OFP1 has been shown to regulate *GA20x1* expression and ATH1 was shown to regulate *FLC* expression in previous reports [38], although it is unclear whether OFP1 interaction with ATH1 mediated these effects.

Our data show *35S:OFP1* also increased *FLC* expression level and that *FLC* was repressed in *Atofp1* lines, implying that OFP1 might positively regulate *FLC. FLC* is known to be an important flowering repressor [32]. Therefore, the *At35S:OFP1* late-flowering phenotype may be at least partly caused by increased *FLC* expression level. In particular, the ATH1 overexpression result in higher *FLC* expression, therefore ATH1 can delay flowering. However, ATH1 as a transcription repressor, it implies that the regulation of ATH1 activating *FLC* possible is not direct. At the same time, the results of this work show that ATH1 could decrease *GA20x1* expression level, while *ath1* exhibited the reverse result. Furthermore, ATH1 might repress stem elongation by decreasing *GA20x1* expression level, it is an important enzyme for GA biosynthesis. Indeed, in previous reports GA had been demonstrated to play a crucial role in stem development [39,40].

As a final observation, *Atofp1* lines showed only partial effects on flower basal boundary formation, as similarly observed for *Atath1* function. This result further implies that OFP1 and ATH1, via a protein–protein interaction, might share a common pathway to influence flower development. However, *Atofp1* lines did not show any distinct effects on development of basal boundary development in other organs. Collectively, these results suggest that ATH1 protein or OFP1-ATH1 complexes possibly regulate flower development via interactions.

In summary, data described in this work support a model that ATH1-OFP1 protein complexes form through protein interactions. Moreover, ATH1-OFP1 can regulate flowering time, repress stem elongation, and control flower basal boundary development. Moreover, these functions are possibly partly carried out by regulation of *FLC* and *GA20x1* expression levels. At the same time, the regulatory functional mechanism of OFP1-ATH1 is complicated and diverse due to the fact that OFP1 might depend on ATH1 in order to regulate various biological functions. These results also imply that the protein complex of ATH1-OFP1 possibly contains unknown KNOX proteins or other proteins. The fact that OFP1 may increase the repression function of ATH1 might result from OFP1 negative regulation of the function of ATH1-KNOX complexes, as similarly observed for negative regulation of KNAT7 by OFP4 during formation of the secondary cell wall [30]. As a final note, of the 18 members of the OFP family, only OFP1 has been shown to interact with ATH1, which suggests that the interaction of ATH1 with OFP1 may play a unique and important role in plant growth and development.

## Figures and Tables

**Table 1:** Statistical analysis of different lines (Different letters represent significant differences in the same column. Number of leaves at flowering, plastochron and inflorescence length in T3 progeny of overexpressing lines, mutant lines etc. F-test *p*-values show distinctness among different genetics materials. 6–10 plants were analyzed for each line. ± represent standard deviation).

	Leaf Number at Flowering	Plastochron Day/Leaf	Inflorescence Height (cm)
Col	13.7 (±0.73) e	1.45 (±0.14) bc	8.7 (±0.62) d
*ofp1*	11.8 (±0.62) f	1.49 (±0.10) ab	9.6 (±0.83) c
*ath1*	10.1 (±0.81) g	1.42 (±0.21) c	10.3 (±0.63) b
*35S:OFP1*	15.5 (±0.72) c	1.43 (±0.09) c	7.1 (±0.62) f
*35S:ATH1*	17.1 (±0.80) a	1.52 (±0.11) a	1.4 (±0.62) h
*35S:OFP1/ath1*	15.1 (±0.67) d	1.45 (±0.15) bc	8.1 (±0.81) e
*35S:ATH1*/*ofp1* *ath1 ofp1*	16.3 (±0.79) b 9.9 (±0.87) g	1.49 (±0.14) ab 1.41 (±0.20) c	2.9 (±0.86) g 10.7 (±0.91) a

## Supplementary Materials

The following are available online at http://www.mdpi.com/2073-4425/9/8/399/s1, Figure S1: Additional negative control and positive control are shown. Figure S2: *35s:ATH1*, *35s:ATH1*/*ofp1*, *ath1 ofp1* and other lines are shown, File S1: Primers description.
