# Supplementary material for: OFP1 Interaction with ATH1 Regulates Stem Growth, Flowering Time and Flower Basal Boundary Formation in Arabidopsis
Source: Genes (Basel). 2018 Aug 6;9(8):399. doi: 10.3390/genes9080399 (PMC6116164; doi:10.3390/genes9080399)
Supplement: Supplementary file 1 [file genes-09-00399-s001.zip › File S1.pdf]

1. Atath1 was created and confirmed :

AtATH1 5′-TGGAACATAGAGGTTTCT TGAGTC-3′,

5′-TCTATCGATGTTCAAGATAACCGC-3′

T-DNA primer 5′-GTGGATTGAT GTGATAT CTCC-3′

2. DNA fragments containing the homeodomain (HD) and SKY-BELL domain of ATH1

HD 5′-CTCCACGGTCTATCGATGTTCA-3′,

5′-CCCCACATGTT GTTTGAGGAA-3′;

SKY-BELL 5′-TCGGTGAAGAAGATTTCCCGTT-3′,

5′-TGATGCAAGA CTCAATGAAAGCTC-3′

3. Clone of *OFP1* and *ATH1*

ATH1 5′-TCCTCCACTTCATCCTTTGG-3′

5′-CGTTGGGTTGAATGTGACTG-3′

OFP1 5′-ATGGGT-AATAACTATCGGTTTAAGCT-3′

5′-TTATTTGGAATGGGGTGGTGGAAGA-3′

4. qRT-PCR assay(5′-3′)

APT: TCCCAGAATCGCTAAGATTGCC, CCTTTCCCTTAAGCTCTG

GA2ox1：GTGTCGTACGAGGTGTTGGAGA , CCAAGTCTATAATCACCA AGC

FLC：ACCTTCTCCAAACGTCGCAA，CGGAGATTTGTCCAGCAGGT

ATH1：AGGCGGGTTTCG GATCTACAT, TTATTTATGCATTGCTTGGCT

OFP1：GAAACACCACGCAGTCCCTA, GGCGTAAAGGTTGCGGTTTT

*ACT1:*CCAGAAGGATGCATATGTTGGTGA,GAGGAGCCTCGGTAAGAAGA
