# Supplementary figures and images for: OFP1 Interaction with ATH1 Regulates Stem Growth, Flowering Time and Flower Basal Boundary Formation in Arabidopsis
Source: Genes (Basel). 2018 Aug 6;9(8):399. doi: 10.3390/genes9080399 (PMC6116164; doi:10.3390/genes9080399)

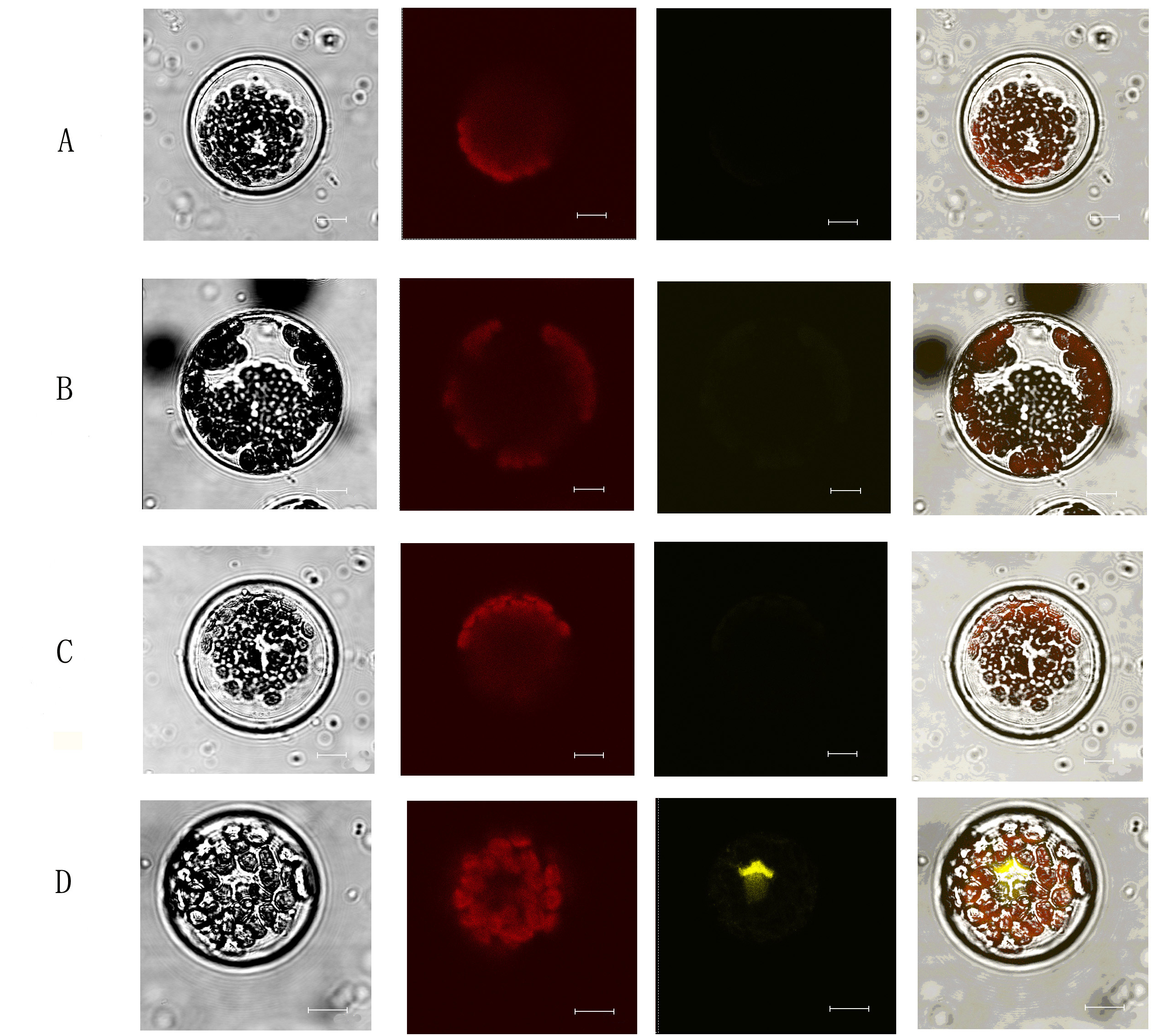

Supplement: Supplementary file 1 [file genes-09-00399-s001.zip › Figure S1.jpg]

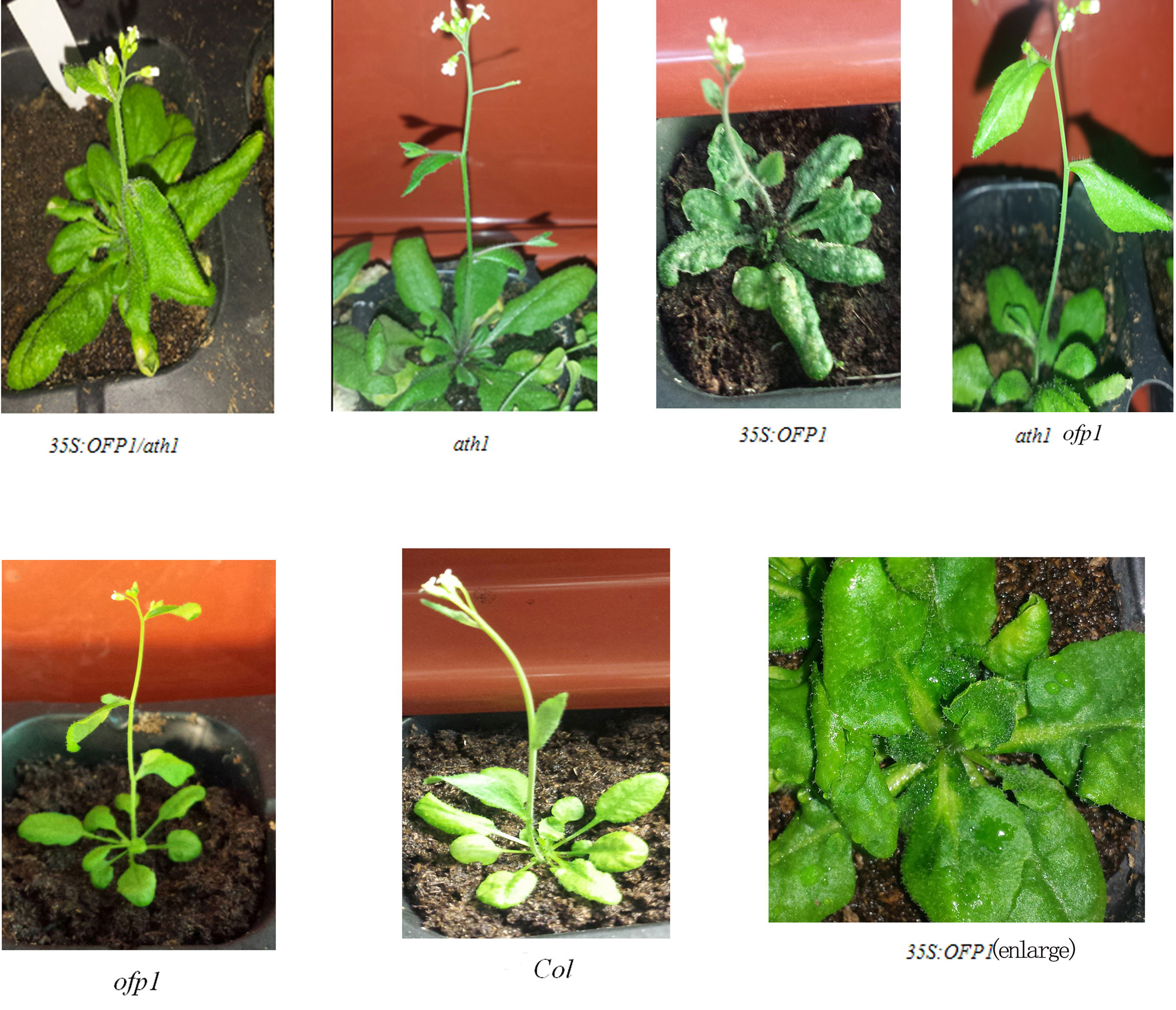

Supplement: Supplementary file 1 [file genes-09-00399-s001.zip › Figure S2.jpg]
